# “It’s my business, it’s my body, it’s my money”: experiences of smokers who are not planning to quit in the next 30 days and their views about treatment options

**DOI:** 10.1186/s12889-016-3395-0

**Published:** 2016-08-04

**Authors:** YK Bartlett, N. Gartland, A. Wearden, CJ Armitage, B. Borrelli

**Affiliations:** 1 Manchester Centre for Health Psychology, School of Psychological Sciences, Manchester Academic Health Science Centre, University of Manchester, Manchester, UK; 2 Henry M Goldman School of Dental Medicine, Boston University, Boston, MA USA

**Keywords:** Smoking, Motivation, Cessation, Focus groups

## Abstract

**Background:**

Current evidence-based smoking cessation treatments in the UK are only offered to smokers ready to quit within 30 days. This study reports the experiences of smokers who are not ready to quit and explores the types of intervention approaches that might engage them.

**Methods:**

Five focus groups were conducted with smokers who had no plans to quit within 30 days (*n* = 32, 44 % female). Verbatim transcripts were analyzed thematically using Nvivo 10 software.

**Results:**

Participants were ambivalent towards their own smoking, but the majority indicated they would like to quit someday. Smoking was seen both to hinder and facilitate social interactions, depending on the social norms of the participant’s social circle. Participants reported that, when they perceive pressure to quit smoking, they respond defensively; concurrently, existing approaches to encouraging smoking cessation were seen as unappealing. In contrast, the importance of intrinsic motivation to quit was emphasized, and interventions that were tailored, increased intrinsic motivation and kept the smoker engaged in activities incompatible with smoking were preferred.

**Conclusions:**

Despite not planning to quit in the next 30 days, the majority of participants wanted to quit smoking at some point. Even if existing services were offered to smokers not planning to quit in the next 30 days, it is unlikely that these services would meet the needs of this population. Future research should explore novel approaches to appeal specifically to smokers not planning to quit in the next 30 days, such as encouraging engagement with activities incompatible with smoking and fostering non-smoking habits.

## Background

Smoking prevalence in the UK is 18.5 % and has remained between 18 % and 20 % since 2012 [1, 2]. This is despite the introduction of a nationwide ban on smoking in public places in 2007, and the continued provision of evidence-based behavioural and pharmacological support [3, 4] through specialist smoking cessation services [5, 6]. One possible reason for the plateauing in smoking prevalence is that although smoking cessation services can be accessed in a variety of ways (e.g. directly by phone or email, or through a referral from a health care professional (HCP)), they are only free at point of contact for the minority (8 %) of UK smokers who want to quit smoking in the next 30 days [7]. As part of the World Health Organisation’s guidelines for supporting smoking cessation it is recommended that if smokers contact any HCP for any reason then they should be asked about their smoking and offered brief advice [8]. Individual countries have expanded on these guidelines to recommend that HCPs provide motivational counselling, direct smokers to appropriate support services, or provide brief behavior change interventions [3, 9]. Contrary to this WHO guidance, ~49 % of UK smokers who visited a healthcare professional reported not receiving any advice to quit [10].

Findings suggest there are a number of factors that influence HCPs’ decision to raise smoking during a consultation, for example; the anticipated response from the patient, the patient’s perceieved motivation to quit, and whether the consultation is due to a smoking-related health problem [11, 12]. The stepped approach to providing advice and support to smokers has been criticized because the level of intervention provided is heavily influenced by the smoker’s readiness to quit at the time they are speaking to the HCP [13]. Thus, even if providers do raise smoking during the consultation, as there are few options for treatment of smokers who are not ready to quit within 30 days it is currently unclear how these smokers would be engaged in cessation. A qualitative exploration of the experiences, views and ideas of these smokers would help researchers to understand this sub-population of smokers and provide insight into how to engage them in an intervention.

Previous qualitative research has explored the views of different sub-populations of smokers, such as disadvantaged smokers or smokers with mental illness [14, 15]. However, to our knowledge there is only one other focus group study that describes the views of smokers who are not planning to quit in the near future [16]. As part of their sample, Uppal et al. [16] recruited smokers who were not ready to quit ‘in the immediate context’ (p.4) and found that these smokers placed high importance on ‘wanting’ to quit and the presence of cognitive dissonance through the identification of positive and negative attributes of smoking and being a smoker. In addition, those lower in motivation to quit reported they were less likely to contact stop smoking services than those with higher motivation to quit. However, Uppal et al.’s study is limited by not specifying a time limit on ‘the immediate context’ (p. 4), which is important in the context of UK services being offered only to those smokers who do not plan to quit within 30 days. The present study focuses only on smokers who do not plan to quit within 30 days, and also seeks to explore what approaches may be useful in promoting smoking cessation in this population.

The aims of the present study are to answer: (a) what are the experiences of smokers with no plans to quit within 30 days?; and (b) what are these smokers’ views on existing smoking cessation approaches and what might engage them in smoking cessation?

## Methods

### Design

Focus groups were used as they can facilitate a deep understanding of the experiences of a population through the interaction between participants with a shared background [17]. A focus group interview guide was developed by the research team, based on previous work by one of the authors. As recommended in previous literature, the guide consisted of introductory, transition and key questions [18]. See Table 1 for the key questions asked.

**Table 1:** Questions from the focus group schedule

Question type	Questions
Introductory questions	What do you like about smoking?
	What about the other side…what don’t you like about smoking?
	How does it feel to be a smoker in today’s world?
Transition questions	For you, what got in the way of quitting in the past?
	What is holding you back from quitting now?
Key questions	What do you think about the currently available options for quitting smoking?
	a) What are the good things about these options?
	b) What is lacking in these options?
	We would like to develop a programme for smokers who are not ready to quit, what should we include in this programme?
Final question	What do you think are the most important points we should take away from the group to design a smoking cessation intervention for smokers like you

### Participants

Participants were recruited through newspaper advertisements across Greater Manchester, posters, leaflets, online noticeboards/mailing lists, and face-to-face in local shopping areas. Interested participants were screened for inclusion by telephone. Study inclusion criteria were: i) aged 18 years or older, ii) smoked cigarettes in the last 7 days, iii) currently smoke ≥3 cigarettes per day, iv) smoked ≥ 100 cigarettes in their lifetime, and v) did not plan to quit smoking in the next 30 days. The exclusion criteria were: i) unable to speak/read fluently in English, ii) self-reported serious mental illness, iii) self-reported heavy drinking, and iv) current involvement in any other smoking related research. Eligible participants were scheduled for a focus group and sent information sheets and consent forms. The final data set consists of a sample of those who contacted the researchers, were eligible, able to attend a group, and attended the group. Convenience sampling was used, we aimed to recruit both males and females to attend each group.

### Procedure

Focus groups were conducted in Manchester, UK with regular smokers who did not plan to quit smoking within 30 days. The university research ethics committee provided ethical approval. Thematic saturation was reached after five focus groups. Focus groups were held between September 2014 and January 2015 and were conducted in line with recommendations [19]. Participants gave informed consent prior to any research activities. Consented participants completed a background questionnaire, which assessed demographic and smoking-related information. Groups were conducted at the authors’ UK University and lasted between 100 and 120 min (including consenting procedures and questionnaire completion), participants were given a £20 (~$31) gift card and offered refreshments at the group as reimbursement for their time. Three members of research staff attended each group: i) a moderator who guided the group, asked key questions and encouraged discussion between participants, ii) a co-moderator who asked additional questions or probed for further detail, and iii) a note-taker. At the end of each group the moderator provided a summary of the main points, and comments were invited from participants, the co-moderator and the note-taker. Following the group, the moderator, co-moderator and note-taker held a short debriefing where key points made by the group were noted as well as any suggested changes to the questions asked, the order, or the amount of time spent on each to ensure thematic saturation was reached for key questions. Groups were audio recorded, transcribed verbatim and the transcripts anonymized.

### Analysis

Structured analysis followed by interpretation of the themes was used to gain an understanding of the experiences and perceptions of the participants [20, 21]. Anonymized transcripts were analyzed thematically following six steps: i) familiarization, ii) generating initial codes, iii) searching for themes, iv) reviewing themes, v) defining and naming themes, and vi) writing results [22]. Two researchers identified themes and sub-themes independently then formed a consensus on the overall structure of the themes through discussion. As outlined in Braun and Clarke (2006), the aim at this stage of the analysis was to summarise the sub-themes into internally consistent and distinctive themes that would allow for a coherent presentation of the data [22]. Any disagreements that could not be resolved were discussed with a third researcher. Two researchers coded all five transcripts independently according to the agreed thematic structure using NVivo 10 software. Any discrepancies at this stage were resolved through discussion. The content of the themes was summarised and interpreted iteratively within the research team and the final report was checked against the original themes to ensure consistency and that any divergent cases were reported. To enhance the accuracy of interpretation, all researchers involved in the analysis had also been involved in collecting the data [23].

## Results

The final sample included 32 participants who were aged between 20 and 79 years old (*M* = 43.2, *SD* = 18.1) and 44 % (*n* = 14) were women (see Table 2).

**Table 2:** Participant characteristics

Characteristic	Participants *n* = 32
	N (%)/Mean (SD)
Demographics	
Age	43.2 (18.1)
Women	14 (44 %)
Caucasian/White	22 (73 %)
Less than a university level education	16 (53 %)
Employment	
Employed full or part-time	10 (31 %)
Unemployed	9 (28 %)
Student	7 (22 %)
Retired	4 (13 %)
Homemaker	1 (3 %)
Unable to work due to disability or long-term illness	1 (3 %)
Smoking characteristics	
Years smoked	23.8 (16.4)
Smoke within 30 min of waking	17 (53 %)
Previous quit attempt longer than 24 h	16 (50 %)

Eleven sub-themes were identified and structured into three over-arching themes: i) experience of being a smoker; ii) views of smoking in the context of social groups and wider society; and iii) quitting: past attempts and future approaches (see Table 3 for thematic structure). The results are presented under the main theme headings with representative quotations.

**Table 3:** Thematic structure

Themes		Sub-themes
Experience of Being a Smoker		Ambivalence towards smoking
		Perceptions of self as a smoker
Views of Smoking in the Context of Social Groups and Wider Society		Social influence and interference
		Autonomy
Quitting: Past Attempts and Future Approaches	Plans to quit and past experiences of quitting	Perceived effects of quitting
		Views of quit methods
		Information needs
	Suggestions for a smoking cessation program for smokers who are not ready to quit.	Intrinsic motivation
		Individualisation
		Substitution
		Using technology to quit

### Experience of being a smoker

Very few participants were wholly positive or wholly negative about continuing to smoke, but rather the majority of participants expressed ambivalence towards their continued smoking. Reasons for smoking included habit, boredom and enjoyment, whereas health effects, financial cost and smell were identified as negative aspects of smoking. Participants often stated that smoking was something they did without much thought.

‘*You*’*re just that used to opening the packet and just lighting it. It just becomes a habit*… *you*’*re just not even realizing* [*you*’*re*] *lighting another one*.’ Female, aged 33.

The balance between positive and negative views about smoking was different for each individual, and was described as influencing their current motivation to quit smoking.

‘*The enjoyment of cigarettes*, *at the moment it outweighs any constant health considerations*.’ Male, aged 69

Some participants said that if they experienced negative impacts of smoking, they might be more inclined to change:

*I think conversely one of the things that stopped me from having a real go at stopping is that I*’*ve never had a bad cough or anything*’ Male, aged 71.

The above views were expressed in a passive way, however some participants expressed strong negative emotions towards themselves, such as anger and shame, due to the conflict of continuing to smoke while being aware of the negative impacts.

‘[*My father had*] *been debilitated by smoking*…[*the family*] *all smoked while he was there on the bed*, *dying of smoking. It*’*s a form of insanity*.’ Male, aged 52.

In contrast, a minority of participants expressed positive emotions towards themselves such as pride in being rebellious or free.

‘[*Smoking is*] *like you have the freedom to do whatever you want*.’ Female, aged 23.

In both cases the risk inherent in smoking seems to be acknowledged. In the former, the participant sees themselves as illogical for taking this risk (and therefore expresses anger); in the latter, the participant sees themselves as a risk taker and therefore their position as a continuing smoker does not cause any negative emotions.

### Views of Smoking in the Context of Social Groups and Wider Society

Several younger participants mentioned the social aspects of smoking had been strengthened since smoking had been banned in enclosed public places. This was due to smokers being grouped together more often to smoke. However, participants who were older described smoking as an anti-social activity, one that took them away from their friends, or was looked down on.

‘*Well for me*, *personally*, *as a student*, *it*’*s actually very acceptable to be a smoker*.’ Male, aged 24

‘*I*’*m outside having a ciggie*, *and can*’*t mix with decent society*!’ Male, aged 69

Wider societal perceptions of smoking were also discussed. For example, in each group, participants mentioned how the general public’s perception of smoking has changed over time. Some participants expressed frustration that cigarettes had been advertised freely, and were so widely used when they were growing up. Others expressed frustration that society had become more negative towards smoking, and judgemental of those who smoke.

‘*It*’*s hard now*, ‘*cause everywhere is no smoking areas*…*you*’*re stigmatized. You feel stigmatized anyway*.’ Male, aged 53

However, while the perceived stigmatization of smoking was seen as negative, it rarely motivated participants to want to quit smoking, and in fact was acknowledged as counterproductive, making them want to smoke more.

‘*The more the media try to bully me into giving up*, *the more I feel*, *sod them*^1^*I*’*m not going to do it*.’ Male, aged 69

This was sometimes expressed as a statement of the participants’ autonomy in their choice to smoke:

‘*It*’*s my business*, *it*’*s my body*, *it*’*s my money*.’ Female, aged 79

Smoking cessation policies introduced by the government were seen to perpetuate a negative view of smoking and were met with skepticism by some participants. Many mentioned the large amount of tax paid on cigarettes, and questioned whether the government is truly motivated to reduce smoking rates.

‘*It produces so much revenue for the government*, *so they have got to be careful* [*encouraging cessation*].’ Male, aged 64

Participants who felt stigmatized for smoking had negative feelings towards non-smokers and the government; contending that the stigmatization decreased (rather than increased), their motivation to quit. The negative feelings expressed suggest participants recognised non-smokers and governmental campaigns as threatening to their position as continuing smokers who do not plan to quit in the next 30 days and could therefore be reacting defensively.

### Quitting: Past Attempts and Future Approaches

#### Plans to quit and past experiences of quitting

Very few participants indicated they enjoyed smoking and planned to continue, but many stated they would like to quit but did not think they could.

‘*I don*’*t know*, *basically*, *I just don*’*t think I can stop*, *although I really want to try*, *eventually*.’ Male, aged 53

Some individuals provided examples of events or situations that might motivate them to quit in the future, including diagnosis of a health condition, giving up smoking for their children or quitting smoking after completing their their university education. For others, future plans to quit were based on more general intentions and feelings that they ‘should quit’ for their health.

Almost all the participants reported making at least one previous quit attempt (although in the background questionnaire, only half of the participants reported that they had made a previous quit attempt lasting more than 24 h). Some individuals were very positive about their previous quit attempts, recommending treatments they had used to other group participants. However, a previous quit attempt that was perceived as successful conversely made some participants believe that they could quit at any time.

‘*The trouble is*, *because I don*’*t find it a great strain to stop*, *I think*, *oh I can stop*, *so I start again*.’ Male, aged 52

We also asked participants about their views of currently available options for quitting smoking. Most participants had views regarding nicotine replacement therapies (NRT). Price was frequently mentioned as a barrier to procuring these treatments, although it was argued by others that the money saved from not buying cigarettes could be used to purchase NRT. In some groups, participants were unsure whether NRT was available through the National Health Service (NHS) or which NRT treatments were available^2^.

E-cigarettes were discussed by each group without prompting by the moderator. Some participants were skeptical about them, believing the lack of endorsement by the NHS was evidence that e-cigarettes were no better for one’s health than tobacco cigarettes. Others viewed e-cigarettes very positively and disagreed with proposals to ban them in public places, arguing that it is the closest substitute for smoking and should be promoted as a tool for helping people quit smoking. Participants called for more research and clearer advice about the safety of e-cigarettes. Some participants viewed replacing smoking with either NRT or e-cigarettes as not ‘fixing the problem’ of nicotine addiction, and there was uncertainty about whether this would benefit health compared to continued smoking.

On the whole, smoking cessation advertisements were not thought to motivate quit attempts. Some participants thought shocking advertisements would help smokers who are not motivated to quit smoking:

‘*I think you need actually something*…*either something really shocking or like something worthwhile*, *to make you want to do it* [*quit smoking*].’ Female, aged 21

The majority of participants believed current advertisements had no effect on motivation to quit because they reiterated well known information (e.g., health effects of smoking).

‘*It*’*s just making you think*, *well that*’*s disgusting I*’*m going to turn that off. I want to give up*, *I know it*’*s bad for me*, *you don*’*t need to tell me that. Tell me how to give up*, *tell me easy ways*, *tell me good ways of giving up*.’ Male, aged 21

#### Suggestions for a smoking cessation program for smokers who are not ready to quit

In general, participants did not believe external influences could directly affect their motivation to quit smoking. The importance of intrinsic motivation for quitting was emphasized in every group. Some participants argued that intrinsic motivation and willpower alone would be sufficient to quit smoking; for others, intrinsic motivation was seen as a necessary addition to other smoking cessation approaches. A few participants argued that, because they did not want to quit, no program would be able to help them until this changed.

‘*I*’*m going to stop on my own because I have the willpower to do it or I*’*m just going to keep smoking*.’ Female, aged 23

However, participants did describe external influences affecting smoking behavior. For example, participants stated that when in a non-smoking situation (e.g. on an airplane) or performing particular tasks and activities where they habitually did not smoke (e.g. sports) they did not experience nicotine cravings.

‘I’m a godfather, and when I go round to their house, obviously it’s a non-smoking household, but I don’t feel the need to smoke. Once I’m in that space I know I can’t smoke, so there’s almost like this little switch goes on in my head that says, you can’t smoke, no need to smoke.’ Male, aged 43

Furthermore, participants described changing the patterns of their smoking depending on living arrangements and jobs.

‘*The only reason I had quit smoking was because the entire environment around me*-*I was so busy*, *and the people around me were just non*-*smokers*.’ Male, aged 22

In general, participants did not believe these short-term environmental changes would influence their motivation to quit or reduce their smoking in the long-term because returning to situations associated with smoking would trigger a relapse to their original smoking habits. There was a sense that participants were waiting for a *magic bullet* to provide an extra push for them to quit smoking, with some describing this as needing to really want to (intrinsic motivation) whereas others thought if smoking cessation interventions were more shocking this would provide the push needed.

When participants were asked about what kinds of programs might engage smokers who are not ready to quit, some participants suggested that keeping smokers busy and engaged, both mentally, and in terms of doing something with their hands may be an effective approach. This was conceptualized not as a substitution for smoking, but rather as a method of distracting someone from smoking.

‘*So I know the health issues and financial issues don*’*t really matter to people who smoke. So I think making a person feeling* [*sic*], *or getting them busy into something else which makes them feel better*, *that*’*s all*.’ Male, aged 22.

Some participants suggested financial incentives such as money, vouchers or discounted gym membership as a reward for smoking cessation. However, there were concerns raised that once the rewards stopped, people would start smoking again and that if cessation was not verified, people would claim the rewards without changing their smoking behavior.

Participants in each group were clear that any smoking cessation program designed for smokers like them would need to be individualized or tailored to be effective, respecting differences in reasons for smoking, smoking history, interests and social context. Furthermore, personal choice in intervention content was seen as necessary to get people interested, allowing individuals to tailor the programme themselves.

‘*I think that it does need individual treatment*, *because we are all individuals*.’ Male, aged 71.

In terms of accessing smoking cessation programmes some participants mentioned they would like to attend a group, but others thought it would take up too much time. One participant mentioned that they had used a mobile phone application (‘app’) during a previous quit attempt; some thought they would like a smoking cessation app because it would be available whenever they needed it, while others thought it would be too easy to switch off. The frequency with which individuals wanted to interact with a programme varied, with some expressing a desire for frequent meetings, reminders or messages whereas others thought this would become annoying or even counter-productive as it may remind them to smoke.

## Discussion

This is the first study, to the authors' knowledge, to have explored the experiences and views of smokers who are not eligible to receive treatment from UK specialist smoking cessation services (i.e., not planning to quit in the next 30 days). The key findings were that smokers not planning to quit in the next 30 days were ambivalent about their continued smoking but that current approaches to encouraging smoking cessation fail to meet their needs. An individualized approach that keeps smokers busy has the potential to engage this group. The following discussion considers the theoretical and public health implications of the work.

The feeling of ambivalence towards smoking and the individual pros and cons identified by our groups are similar to those that have been identified in smokers with a wide range of levels of motivation to quit [24]. That these have been found in this population of smokers who are not ready to quit within 30 days could indicate that smokers’ feelings towards smoking, and themselves as smokers may not be associated with their readiness to quit smoking. A minority of participants were ‘pro-smoking’, and therefore not motivated or ready to quit at any point, however the majority of our participants indicated a desire to quit someday, but not in the immediate future.

Some participants indicated they did not think they would be able to quit, which in turn, had an impact on their motivation and readiness to make a quit attempt. Other participants reported that their high confidence in their ability to quit meant there was no need to immediately quit smoking. This was described as the result of previous quit attempts that were perceived by participants as successful. As all participants were current smokers it would be interesting to explore further how previous quit attempts are perceived as successes or failures by continuing smokers with no immediate plans to quit. Self-efficacy has previously been identified as a key component of reduction in smoking [25], avoiding relapse [26], and a mediator of smoking cessation [27]. However, our qualitative findings indicate that high self-efficacy may only be beneficial if accompanied by high motivation and readiness to quit. It could be suggested that those with high self-efficacy but low motivation and low readiness to quit may feel confident they would be able to quit smoking, but still do not expect to quit in the near future. This study did not ask explicitly about participants’ expectations in terms of quitting smoking, but expectations have been found to be a better predictor of behavior change than intentions (even when controlling for self-efficacy, and the effects of past behavior), potentially because asking about expectations elicits more reflective processing [28]. Future research could explore the relationships between self-efficacy, motivation, readiness to quit, intentions and expectations in more detail to ascertain whether expectations to quit differ in smokers who express a desire to quit, but have no immediate plans to do so.

For many participants, smoking was seen as an anti-social activity, which took them away from their friends. However, amongst some participants (particularly students aged in their 20s), smoking was seen as a social activity. Previous research has indicated that students perceive health messages around smoking as appropriate for teens and older adults, but not for themselves [29]. Future interventions could explore targeting content for social groups to take account of different social norms.

Some participants perceived reduced societal acceptance of smoking, through both governmental campaigns and the opinions of non-smokers. This was seen to increase the perceived pressure to quit smoking. However, rather than increasing their motivation or readiness to quit, it was reported that this perceived pressure produced a counter-productive defensive response. When discussing smoking cessation campaigns in the media it is interesting that participants focused only on the negative campaigns (those that focus on the negative health effects of smoking). In the UK, between 2005 and 2010 44 % of campaigns were negative, whereas 52 % of campaigns were positive (focused on hope and happiness as a result of quitting), the remainder being neutral [30]. This could indicate that although participants reported the campaigns were not effective, the negative campaigns did result in an emotional response and increased discomfort between processing the messages and continuing to smoke (making them more memorable). Self-affirmation (encouraging individuals to elaborate on values that are important to them) has been found to reduce this defensiveness in response to graphic warnings on cigarette packets [31] and amongst smokers with low socio-economic status [32] and could have the potential to decrease defensive processing in smokers who are not ready to quit. It could also be the case that amongst a peer group of smokers, participants were unwilling to admit the effects these campaigns have had on their smoking. While this cannot be discounted, all participants were current smokers, so even if they have had more of an effect than disclosed in the groups, they have not been effective in terms of encouraging and supporting these participants to quit.

Participants reported confusion and misinformation around the currently available options for quitting smoking. There was clear interest in e-cigarettes (evidenced by the topic being spontaneously broached by each group), but there was little knowledge of how continued use of nicotine compares with continued smoking in terms of health effects. The confusion associated with different approaches to quit meant participants were less likely to want to use interventions such as NRT. A recent review published by Public Health England [33] aimed to address the need for clear evidence related to e-cigarettes. However, the report has generated controversy [34–36]. Therefore the need for clarification of the role e-cigarettes could play in smoking cessation is ongoing for smokers, non-smokers, and the HCPs who support smoking cessation.

Participants acknowledged that their smoking was often habitual and was not necessarily motivated by a desire to smoke, but was triggered by being in a circumstance where one usually smokes. Conversely, there were circumstances identified where the habitual response was not to smoke. This can be understood in terms of participants reacting to cues from their environment. Situational cues to smoking have formed part of smoking models for many years, and these include internal cues such as negative affect and external cues such as drinking alcohol (see [37] and [38] as examples). Previous research has found that smokers are less likely to smoke while working (than at leisure) and more likely to smoke when they were inactive and described themselves as ‘between activities’ [39]. Participants in the current sample recognised that ‘keeping busy’ could be an effective approach to encouraging cessation.

The idea of keeping busy, in combination with the description of tasks and activities that participants undertook where they habitually did not smoke (e.g. participating in sports) could suggest that encouraging smokers to increase the amount of time spent doing activities that are not associated with smoking may help increase the time between cigarettes, and encourage the formation of ‘non-smoking habits’ in those who are not ready to quit at present. It could be speculated that this approach could have the potential to be more acceptable to individuals who are not ready to quit than decreasing environmental cues to smoke (such as removing ashtrays from the home) as it is less focused on smoking related behavior (which an individual may be unwilling to target), and more focused on non-smoking behavior, which may be less likely to provoke a defensive response. Previous work has utilized situational cues in a number of ways. For example, successful non-smoking habit formation has been encouraged by asking participants to link smoking situations with non-smoking solutions in if-then plans (e.g. ‘If I am tempted to smoke when I need a lift, then I will do something instead of smoking’) [40]. Additionally, it has been suggested that situational cues for smoking could be utilised to identify situations where someone is at risk of relapse to enable provision of tailored ‘just in time’ interventions to those who have quit smoking [41]. The present research could suggest that encouraging smokers to engage in activities not associated with smoking, thereby utilising situational cues associated with non-smoking may present an opportunity to engage people who do not want to quit smoking within 30 days.

### Strengths and limitations

The present research included a wide range of recruitment approaches, which resulted in a diverse population in terms of smoking history, age and employment status. This diversity led to lively discussion between participants during the focus groups, and a wide range of views expressed. It also highlighted the need for smoking cessation approaches for this population to be tailored, and for the diversity within smokers who do not want to quit within 30 days to be recognised. As all the participants were current smokers who were not ready to quit this seemed to allow for open discussion without fear of judgement. Conversely however, this similarity may have led to fear of judgement when voicing anti-smoking views. The range of opinions given in the groups suggests this was not the case, but the findings should be interpreted with this potential in mind. The screening process successfully identified smokers who were not planning to quit within 30 days in all but one case. One participant became ready to quit smoking between the phone screening and group attendance; in the future, screening could be repeated on the day of the group to avoid this. In addition, the present convenience sample was self-selected: The information sheet outlined the purpose of the research and therefore may have resulted in greater attendance by those who had strong opinions either about continuing to smoke, or about quitting, and were willing to share them in a group. Finally, the authors who worked on the analysis had all attended at least one of the focus groups, while this provided valuable context to the interpretation [23], a researcher with no prior knowledge of the research aims or groups may have been able to provide a different perspective.

## Conclusions

Many smokers who are not planning to quit in the next 30 days would like to quit at some point. Current smoking cessation services are not available to this population of smokers, and interventions that could provide support outside of a HCP consultation should be explored. This research explored the views of smokers who are not planning to quit within the next 30 days and found that this population of smokers reported a lack of information about available treatment options, however, until they become more ready to quit it is unlikely that they would seek this information independently. Therefore, novel methods for delivering this information in a way that does not increase perceived pressure to quit are needed. One possible way to do this is to focus on activities or situations that are not associated with smoking to try and encourage breaking habits around smoking and, forming habits that are not associated with smoking.

## Abbreviations

HCP, Health Care Professional; NHS, National Health Service; NRT, Nicotine Replacement Therapy

## Footnotes

1 Dismissive colloquialism

2 In the UK, NRT is available by prescription and obtained at no cost if individuals are on a low wage, receiving benefits, or have certain health conditions

## References

[1] Office for National Statistics. Integrated household survey, January to December 2014. http://tinyurl.com/zrpcw3o. Published October 2015. Last accessed July 2016

[2] Smoking in England. Top line findings from Smokers toolkit Study (ref: STS140721). Published online: 15/06/16. Retrieved 17/06/16 from http://www.smokinginengland.info/latest-statistics/

[3] Fiore M, Jaén CR, Baker TB, et al. A clinical practice guideline for treating tobacco use and dependence: 2008 update. A US Public Health Service report. Am J Prev Med. 2008; doi:10.1016/j.amepre.2008.04.009. 10.1016/j.amepre.2008.04.009 PMC4465757 18617085

[4] National Institute for Health and Care Excellence (NICE). NICE guidelines [PH10]: Smoking cessation services. https://www.nice.org.uk/guidance/ph10. Published February 2008. Last accessed July 2016

[5] Brose LS, West R, McDermott MS, Fidler JA, Croghan E, McEwen, A. What makes for an effective steop-smoking service? Thorax. 2011; doi:10.1136/thoraxjnl-2011-200251 10.1136/thoraxjnl-2011-200251 21709164

[6] West R, May S, West M, Croghan E, McEwen A. Performance of English stop smoking services in first 10 years: analysis of service monitoring data. BMJ.2013;.doi: http://dx.doi.org/10.1136/bmj.f4921 10.1136/bmj.f4921 23963106

[7] Office for National Statistics. (2009). Opinions survey report No.40: Smoking-related behaviour and attitudes, 2008/2009. http://tinyurl.com/zsnovup. Published July 2009. Last accessed July 2016

[8] World Health Organization Framework Convention on Tobacco Control. Guidelines for implementation of Article 14: Demand reduction measures concerning tobacco dependence and cessation. http://tinyurl.com/j359snm. Published 2010. Last Accessed July 2016

[9] Bailey V, Soni A, Alessi C, et al. The NHS’s role in the public’s health: a report from the NHS future forum. http://tinyurl.com/mskyhk6. Published January 2012. Last accessed July 2016

[10] Borland R, Li L, Driezen P (2012). Cessation assistance reported by smokers in 15 countries participating in the International Tobacco Control (ITC) policy evaluation surveys. Addiction.

[11] Borrelli B, Hechy JP, Papandonatos GD, Emmons KM, Tatewosian LR, Abrams DB (2001). Smoking-cessation counseling in the home. Attitudes, beliefs and behaviors of home healthcare nurses. J Prev Med.

[12] Coleman T, Murphy E, Cheater F (2000). Factors influencing discussions of smoking between general practictioners and patients who smoke: a qualitative study. Brit J Gen Pract.

[13] Richter KP, Ellerbeck EF. It’s time to change the default for tobacco treatment. Addiction*.* 2015; doi:10.1111/add.12734 10.1111/add.12734 25323093

[14] Wiltshire S, Bancroft A, Parry O, Amos A (2003). ‘I came back here and started smoking again’: perceptions and experiences of quitting among disadvantaged smokers. Health Educ Res.

[15] Morris CD, Waxmonsky JA, May MG, Giese AA (2009). What Do Persons with Mental Illnesses Need to Quit Smoking? Mental Health Consumer and Provider Perspectives. Psychiatr Rehabil J.

[16] Uppal N, Shabab L, Ratschen BJ (2013). The forgotten smoker: a qualitative study of attitudes towards smoking, quitting, and tobacco control policies among continuing smokers. BMC Public Health.

[17] Morgan DL, Kruegar RA, Morgan DL (1993). When to use focus groups and why. Successful focus groups: Advancing the state of the art.

[18] Krueger RA (1997). Developing questions for focus groups (Vol. 3).

[19] Morgan DL, Krueger RA (1997). The Focus Group Kit Volumes 1–6.

[20] Miles MB, Huberman AM (1984). Qualitative data analysis: A sourcebook of new methods.

[21] Gibbs GR (2008). Qualitative data analysis: Explorations with Nvivo.

[22] Braun V, Clarke V. Using thematic analysis in psychology. Qual Res Psychol. 2006; doi:10.1191/1478088706qp063oa

[23] Knodel J, Morgan DL (1993). The design and analysis of focus group studies: A practical approach. Successful focus groups: Advancing the state of the art.

[24] Velicer WF, Prochaska JO, Diclemente CC, Brandenburg N. Decisional balance measure for assessing and predicting smoking status. J Pers Soc Psychol. 1985; doi:10.1037/0022-3514.48.5.1279 10.1037//0022-3514.48.5.1279 3998990

[25] Cupertino AP, Berg C, Gajewski B, Hui SKA, Richter K, Catley D, et al. Change in self-efficacy, autonomous and controlled motivation predicting smoking. J Health Psychol. 2012; doi:10.1177/1359105311422457 10.1177/1359105311422457 PMC3549683 22076554

[26] Elfeddali I, Bolman C, Candel MJJM, Wiers RW, De Vries H. The role of self-efficacy, recovery self-efficacy, and preparatory planning in predicting short-term smoking relapse. Brit J health Psych. 2012;.doi:10.1111/j.2044-8287.2011.02032.x 10.1111/j.2044-8287.2011.02032.x 22107073

[27] Borrelli B, Mermelstein R (1998). The role of weight concern and self-efficacy in smoking cessation and weight gain among smokers in a clinic-based cessation program. Addict Behav.

[28] Armitage CJ, Norman P, Alganem S, & Conner M. Expectations are more predictive of behavior than behavioral intentions: Evidence from two prospective studies. Ann Behav Med 2015**;** doi:10.1007/s12160-014-9653-4 10.1007/s12160-014-9653-4 25623893

[29] Staten RR, Ridner SL. College students’ perspective on smoking cessation: “If the message doesn’t speak to me, I don’t hear it”. Issues Ment Health Nurs 2007; doi:10.1080/01612840600997990 10.1080/01612840600997990 17130010

[30] Richardson S, Langley T, Szatkowski L, Sims M, Gilmore A, McNeill A & Lewis S. How does the emotive content of televised anti-smoking mass media campaigns influence monthly calls to the NHS Stop Smoking helpline in England? Prev Med 2014; doi:10.1016/j.ypmed.2014.08.030 10.1016/j.ypmed.2014.08.030 PMC4262576 25197004

[31] Harris PR, Mayle K, Mabbott L, Napper L. Self-affirmation reduces smokers’ defensiveness to graphic on-pack cigarette warning labels. Health Psychol. 2007; doi:10.1037/0278-6133.26.4.437 10.1037/0278-6133.26.4.437 17605563

[32] Armitage CJ, Harris PR, Hepton G, Napper L. Self-affirmation increases acceptance of health-risk information among UK adult smokers with low socioeconomic status. Psychol Addict Behav. 2008; doi:10.1037/0893-164×.22.1.88 10.1037/0893-164X.22.1.88 18298234

[33] McNeill A, Brose L, Calder R, Hitchman S, Hajek P, McRobbie H. E-cigarettes: and evidence update. A report commissioned by Public Health England. http://tinyurl.com/qyy3rey. Published August 2015. Last accessed July 2016

[34] McKee M, Capewell S. Evidence about electronic cigarettes: a foundation built on rock or sand? BMJ. 2015; doi: http://dx.doi.org/10.1136/bmj.h4863 10.1136/bmj.h4863 26374616

[35] Public Health England. (2015). E-cigarettes: an emerging public health consensus joint statement on e-cigarettes by Public Health England and other UK public health organisations. http://tinyurl.com/o78ltg8. Published September 2015. Last accessed July 2016

[36] E-cigarettes: Public Health England’s evidence-based confusion [editorial]. The Lancet. 2015. doi:http://dx.doi.org/10.1016/S0140-6736(15)00042-2.

[37] Kassel JD, Stroud LR, Paronis CA. Smoking, stress, and negative affect: correlation, causation, and context across stages of smoking. Psycho Bull 2003; doi:10.1037/0033-2909.129.2.270 10.1037/0033-2909.129.2.270 12696841

[38] Shiffman S, Dunbar M, Kirchner T, Li X, Tindle H, Anderson S, Scholl S. Smoker reactivity to cues: effects on craving and on smoking behavior. J Abnorm Psychol 2013; doi: 10.1037/a0028339 10.1037/a0028339 PMC3988583 22708884

[39] Shiffman S, Gwaltney CJ, Balabanis MH, Liu KS, Paty JA, Kassel JD, Kickcox M & Gnys M. immediate antecedents of cigarette smoking: an analysis from ecological momentary assessment. J Abnorm Psychol 2002; doi: 10.1037/0021-843×.111.4.531 10.1037//0021-843x.111.4.531 12428767

[40] Armitage CJ. A volitional help sheet to encourage smoking cessation: a randomized exploratory trial. Health Psychol 2008; doi: 10.1037/0278-6133.27.5.557. 10.1037/0278-6133.27.5.557 18823182

[41] McClernon FJ, Choudhury RR. I am your smartphone and I know you are about to smoke: The application of mobile sensing and computing approaches to smoking research and treatment. Nicotine Tob Res 2013; doi: 10.1093/ntr/ntt054 10.1093/ntr/ntt054 PMC3768335 23703731

